# Structural Regulation of Mechanical Gating in Molecular Junctions

**DOI:** 10.1021/acs.nanolett.3c00043

**Published:** 2023-05-02

**Authors:** Biswajit Pabi, Jakub Šebesta, Richard Korytár, Oren Tal, Atindra Nath Pal

**Affiliations:** † Department of Condensed Matter and Materials Physics, S. N. Bose National Centre for Basic Sciences, Sector III, Block JD, Salt Lake, Kolkata 700106, India; ‡ Department of Condensed Matter Physics, Faculty of Mathematics and Physics, Charles University, CZ-121 16 Praha 2, Czech Republic; § Materials Theory, Department of Physics and Astronomy, Uppsala University Box 516, 751 20 Uppsala, Sweden; ∥ Department of Chemical and Biological Physics, Weizmann Institute of Science, Rehovot 7610001, Israel

**Keywords:** mechanical gating, molecular junction, transition voltage spectroscopy, orbital hybridization, ferrocene, break junction

## Abstract

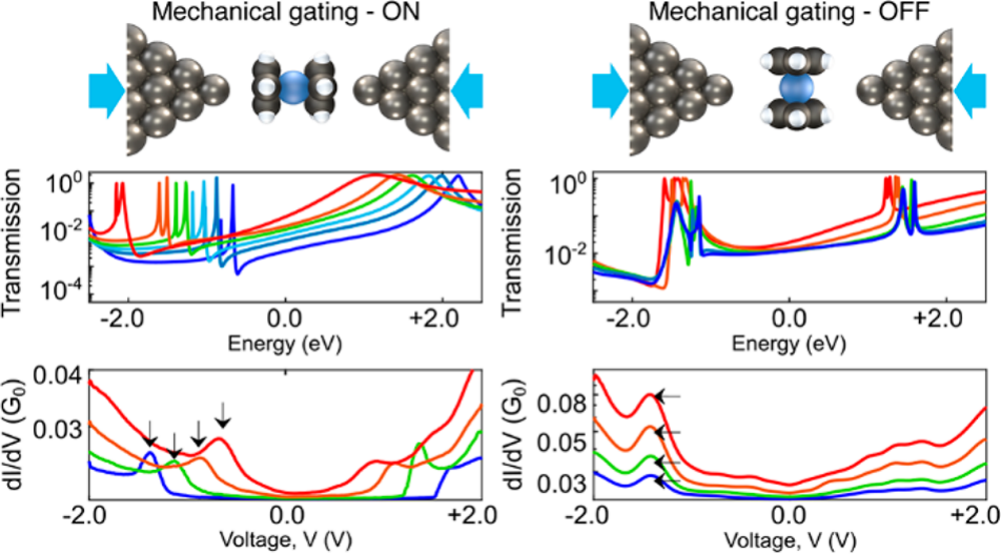

In contrast to silicon-based transistors, single-molecule junctions can be gated by simple mechanical means. Specifically, charge can be transferred between the junction’s electrodes and its molecular bridge when the interelectrode distance is modified, leading to variations in the electronic transport properties of the junction. While this effect has been studied extensively, the influence of the molecular orientation on mechanical gating has not been addressed, despite its potential influence on the gating effectiveness. Here, we show that the same molecular junction can experience either clear mechanical gating or none, depending on the molecular orientation in the junctions. The effect is found in silver–ferrocene–silver break junctions and analyzed in view of ab initio and transport calculations, where the influence of the molecular orbital geometry on charge transfer to or from the molecule is revealed. The molecular orientation is thus a new degree of freedom that can be used to optimize mechanically gated molecular junctions.

One of the fascinating properties of molecular junctions is their ability to function as nanoscale electromechanical devices. In particular, single-molecule junctions allow the study of coupling between mechanical and electronic degrees of freedom in a structure of a typical single-nanometer size that has a dominant quantum nature and a pronounced orbital character. This combination has been used to study diverse phenomena, including electron–phonon interaction,^1−6^ quantum interference^7,8^ and charge reorganization^9^ in the miniaturization limit for electronic conductors. Interestingly, in addition to the more standard electrostatic gating of molecular junctions, these junctions can be mechanically gated. By changing the interelectrode distance in the electrode–molecule–electrode junction, molecular energy levels can be shifted to a lower or higher energy, and charge can be transferred from the electrodes to the molecule or vice versa. Consequentially, the electronic transport characteristics of the junction may change. Mechanically gated molecular junctions have been extensively studied both experimentally and theoretically,^7−15^ for example in the context of nanoscale image charge^9^ and optimization of thermoelectricity.^14^ However, the influence of the molecular orientation on mechanical gating has not been examined. Such an influence can be an attractive route for optimization and regulation of mechanical gating with implications on charge, spin, and heat transport in molecular junctions. Here, we show that the mechanical gating of molecular junctions can be dramatically affected by the molecular orientation, where the same molecular junction experiences either a clear mechanical gating or the absence of such an effect, depending on the orientation of the molecule with respect to the electrodes. By comparison of experiments and calculations, this behavior can be related to the orbital nature at the metal-molecule interfaces, allowing the identification of the necessary conditions for mechanical gating. The reported findings in this letter show that the orientation of the molecule is an important factor for the design of mechanically gated molecular junctions and emphasizes the importance of orbital orientation in the general process of metal-molecule charge transfer.

We studied molecular junctions based on suspended individual ferrocene molecules between two silver (Ag) electrode tips. A break junction setup is used to form in situ these molecular junctions (Figure 1) in a cryogenic environment (∼4.2 K). The ferrocene molecules are introduced from a local heated molecular source into a cold atomic-scale Ag junction during repeated junction breaking and making cycles (see section 1 in the Supporting Information). Measurements of current as a function of applied voltage across the junctions (*I*–*V* curves) for different junction realizations reveal two distinctive cases, denoted here as type 1 and type 2 (Figure 2a,b). The presented *I*–*V* curves were measured following a repeated reduction in the interelectrode separation. Zero displacement defines the interelectrode distance in which the junctions were realized, and a negative value indicates junction squeezing. Several steps can be observed in the *I*–*V* curves, translated in Figure 2c,d, to peaks in the corresponding differential conductance versus voltage (d*I*/d*V*–*V*) curves. As will be further discussed below with the aid of ab initio calculations, the peaks originate from the contributions of molecular orbitals to the conductance. Therefore, shifts in the voltage at which the peaks are observed correspond to shifts in the molecular energy levels, with respect to the Fermi level of the electrodes. Figure 2c reveals that the peaks of type 1 are shifted to a lower absolute value of voltage when the interelectrode separation is reduced. Namely, the molecular level or levels that dominate transport in type 1 are shifted toward the Fermi level when the junction is squeezed. In contrast, in Figure 2d, the peaks for type 2 are not shifted in response to a similar mechanical manipulation. Specifically, the inset of Figure 2c presents a significant peak shift from 1.365 to 0.675 V for a reduction in the interelectrode distance of ∼0.6 Å for type 1, whereas a similar behavior is not seen in the inset of Figure 2d for type 2. This is an indication for mechanically induced molecular energy shifts in type 1 and the absence of this effect in type 2 (see Supporting Information, sections 2 and 3 for more details on types 1 and 2, and the reversibility of the observed level shifts).

**Figure 1:**
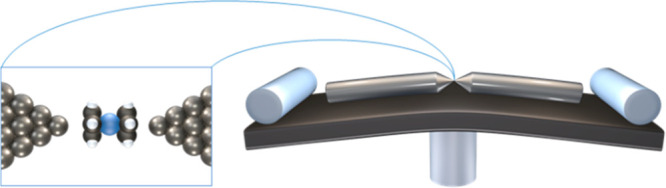
Break junction setup. Schematics of the used break junction setup, in which the distance between the Ag electrode tips can be adjusted in sub-angstrom resolution, and an illustration of a Ag–ferrocene–Ag single-molecule junction.

**Figure 2:**
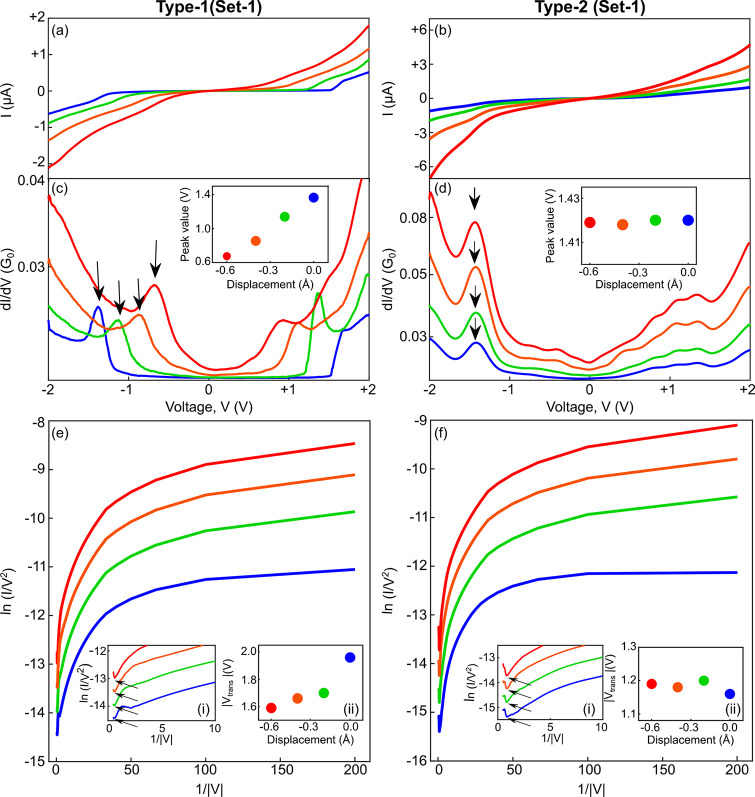
Current–voltage, differential conductance spectra, and transition voltage spectroscopy (TVS) plots. (a,b) Four spectra of current as a function of voltage measured at different interelectrode displacements (for color code and displacement see insets of (c,d) in Ag–ferrocene–Ag molecular junctions with (a, type 1) and without (b, type 2) mechanical gating response. (c) Differential conductance as a function of applied voltage for the junction studied in (a). (d) Same as (c) but with data collected for the molecular junction studied in (b). Insets (c,d): Absolute values of peak position (marked with arrows in (c,d) as a function of interelectrode displacement. (e,f) TVS plots constructed from the same *I*–*V* spectra presented in (a,b), showing ln(*I/V*^2^) versus 1/*|V|* for spectra with (e, type 1) and without (f, type 2) mechanical gating response. For consistency, the negative side of the *I*–*V* curves is considered for TVS analysis. Inset (i): Zoomed view of the TVS plots to better present the change of transition voltage upon squeezing. Inset (ii): Transition voltage (absolute values) as a function of interelectrode displacement for type 1 and 2.

The same effect can also be presented using transition voltage spectroscopy (TVS) plots, where the *I*–*V* characteristics in Figure 2a,b are replotted in Figure 2e,f in terms of ln(*I*/*V*^2^) as a function of 1/|*V*|.^16−20^ These plots are expected to have a minimum at a certain transition voltage, *V*_trans_, whenever the current as a function of voltage evolves from a linear dependence to more than a quadratic dependence.^19^ We note that for metallocene molecules with direct contacts to the electrodes (namely, no anchoring side groups are used), the transition voltage dependence on the energetic difference between the electrodes’ Fermi level and the closest molecular energy level(s) is to date unknown. However, regardless the exact dependence, shifts in the transition voltage serve as an indication for shifts in the energy of the molecular levels that dominate electron transport and/or systematic variations in their coupling to the continuum states of the electrodes (i.e., electrode–molecule coupling).^21,22^ As seen below using calculations, we expect dominant level shifts and moderate variations in electrode–molecule coupling. This is manifested as significant shifts in the calculated transmission peaks with generally modest variations in the peak widths. The TVS plots in Figure 2e,f show a clear characteristic minima, with a corresponding transition voltage. The response of the transition voltage to changes in the interelectrode distance is observed in the insets of Figure 2e,f, where a shift is seen for type 1 but not for type 2 (see Supporting Information section 2 for similar data for other junction realizations). We can take advantage of the presence of peaks in the d*I*/d*V* that correspond to molecular levels to shed light on the relation between transition voltage shifts and molecular level shifts. When the interelectrode distance is reduced by 0.6 Å, the transition voltage shifts by 370 mV. Assuming for simplicity a symmetric voltage drop on each electrode–molecule contact, which can be justified as a crude approximation by the roughly symmetric locations of the positive and negative peaks in Figure 2c (see Supporting Information, section 5), the 690 mV shift in the examined peak location in Figure 2c corresponds to a shift of 690/2 = 345 meV of the molecular levels that dominate the electron transport. Note that, for a symmetric voltage drop, the location of the molecular levels relevant for transport with respect to the electrode Fermi level is given by half the voltage at which a peak in the d*I*/d*V* curve appears (this is illustrated below with the aid of calculated transmissions and d*I*/d*V* curves and explained in Supporting Information, section 5). We can conclude that the observed mechanical gating leads to shifts in the transition voltage that are rather similar in magnitude to the roughly estimated shifts in the molecular energy levels (this can also be seen in Supporting Information Figure S2). Our findings show that TVS is a good indicator for level shifts in the examined junction. The rather linear relation between transition voltage shifts and level shifts calls for further study into the mechanistic details behind it, which are beyond the scope of this Letter.

To better understand the nature of types 1 and 2, we turn to density functional theory (DFT) and electron transport calculations (see Supporting Information for details), as presented in Figure 3a–f. For the range of interelectrode displacements that is considered in the experiments, the molecular junction can adapt, according to our calculations, two distinct stable configurations with parallel and perpendicular molecular orientations with respect to the electrode axis, as illustrated in the insets of Figure 3a,b. The calculated total energy as a function of interelectrode separation is presented in Figure 3f for each of these junction configurations. The two energy curves have a clear minimum at different interelectrode separations. At short distances, the perpendicular configuration is energetically preferred, while for longer distances, the parallel configuration is more stable. A similar behavior was also reported for Ag–vanadocene–Ag junctions by Pal et al.^23^

**Figure 3:**
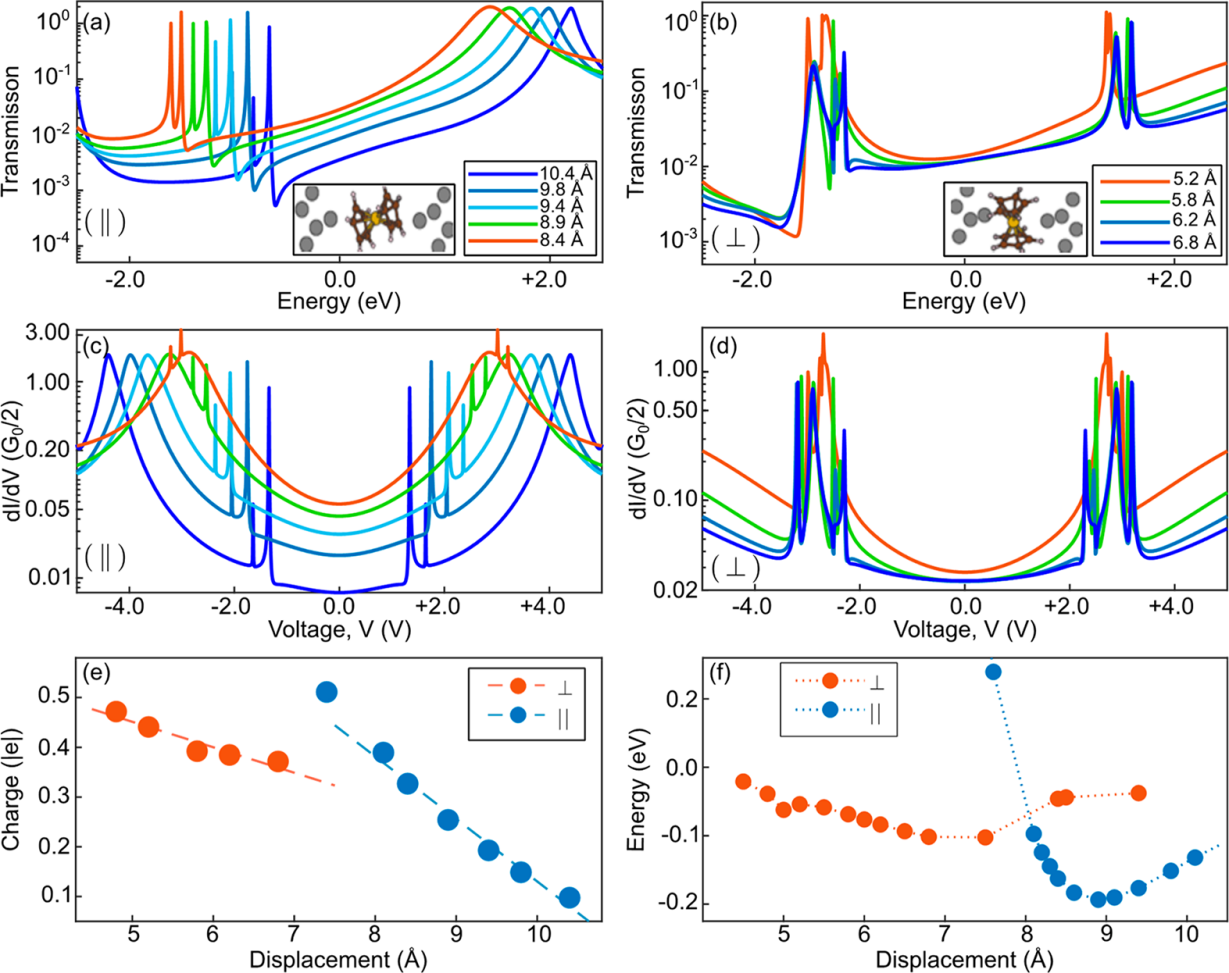
Transport calculations. (a,b) Calculated transmission for parallel (a) and perpendicular (b) molecular orientations in the junction at a varying distance between the electrode tips. Insets: Ball-and-stick models of the calculated structures (only small parts of the electrodes are shown). (c,d) Differential conductance for the parallel (c) and perpendicular (d) configurations at the same varying electrode tip distances as in (a,b). (e) Charging of the ferrocene molecule in the parallel (blue) and perpendicular configurations (orange). (f) Total energy as a function of interelectrode displacement. (⊥,∥) Denotes perpendicular and parallel molecular orientation with respect to the long junction axis.

Figure 3a,b provides the calculated transmission for various interelectrode distances for the two configurations. Here, displacement indicates the distance between the apex atoms of the two electrode tips. The transmissions for the parallel configuration can essentially be understood as being rigidly shifted toward lower energies upon mechanical squeezing. Namely, a mechanical gating is observed, where both the highest occupied molecular orbitals (HOMOs) and the lowest unoccupied molecular orbitals (LUMOs) are shifted to lower energies with no significant changes in the HOMO–LUMO gap.^13^ Below the Fermi energy (taken as zero), pairs of narrow resonances can be seen (HOMOs), while above the Fermi energy, a single broad resonance is found. Its peak transmission is 2, pointing to a two-channel or two-orbital origin (LUMOs). The transmissions for the perpendicular configuration have a richer structure with narrow peaks on both sides of the Fermi energy. Also, the evolution of transmission as a function of stretching is more complex in this case than that for the parallel molecular configuration. However, in contrast to the former case, no clear shifts in the transmission peaks are observed when the interelectrode distance is modified in the given range. Thus, mechanical gating is not found for the perpendicular configuration. We note that in both configurations the transmission resonances are highly asymmetric due to quantum interference,^24^ similarly to the asymmetries reported for molecular junctions based on a ferrocene derivative.^8^

To have a more transparent comparison with the measured data, the described transmission can be directly converted to calculated differential conductance, as presented in Figure 3c,d, assuming a similar voltage drop across the two electrode–molecule contacts (see eq S3 in the Supporting Information). When the molecule is oriented parallel to the junction axis, shifts of peaks are seen, as also found in the experiments for type 1. However, for the perpendicular molecular orientation, a similar effect cannot be found, in agreement with the absence of mechanical gating in the measurements of type 2. Note that the features in Figure 3c,d are sharper than those found in the measured spectra in Figure 2c,d, since the experimental data are widened by the finite temperature and mainly by the lock-in modulation used for differential conductance measurements. An elaborated discussion about the relation between transmission and differential conductance in break junction experiments can be found in the Supporting Information, section 5.

Figure 3e reveals charge transferred from the electrodes to the molecule in equilibrium as a function of interelectrode displacement. The slope is larger in the parallel junction, suggesting that charge reorganization plays an important role in the gating mechanism (see Supporting Information, section 6). The mechanical gating response for the parallel molecule configuration and the absence of this effect for the perpendicular configuration can be ascribed to the orientation of the molecular orbitals that dominate transport with respect to the electrodes and their coupling to the frontier electrode states. Figure 4 presents calculated isosurfaces of the two degenerate LUMOs for an isolated ferrocene, as well as the LUMOs for the parallel and perpendicular junction configurations (calculations were done for a cluster of a single molecule bridging two metal apexes, as explained in the Supporting Information). As can be seen, the junction LUMOs, which are associated with the broad transmission peak above the Fermi energy in the parallel configuration, and the two narrow peaks above the Fermi energy in the perpendicular configuration resemble the LUMOs of the isolated molecule. These orbitals have a significant π-character on the carbon rings; i.e., the wave function spreads away from the ring plane perpendicularly.^25^ The remaining contribution to the LUMOs comes from the Fe 3d atomic orbitals. Therefore, the molecular LUMOs overlap with the electrodes much more efficiently in the parallel configuration than in the perpendicular configuration. This difference makes the LUMO coupling to the frontier electrode states in the parallel configuration more sensitive to changes in the interelectrode distance (seen in Figure 3a as a different width of the broad LUMO peak for different interelectrode distances) than in the perpendicular case. Thus, the orientation of the LUMO with respect to the electrodes affects the mechanical gating efficiency: When the electrodes are pointing to the less-localized part of the LUMO on the carbon ring, mechanical manipulations likely induce orbital modifications and associated charge transfer. In contrast, when the electrodes are pointing toward the more localized part of the LUMO on the Fe ion, mechanical manipulations have a reduced effect on the local orbital structure and the associated charge redistribution. These findings illustrate that molecular orientation, as well as the distribution of molecular orbitals in space are important parameters for the design and control of mechanical gating in molecular junctions and generally for mechanically induced charge transfer in metal–molecule interfaces. Interestingly, based on our analysis, efficient mechanical gating is promoted by delocalization of the frontier molecular orbitals that point toward the electrodes, while for electrostatic gating in single-molecule transistors, efficient gating by a gate electrode is often promoted by more localized orbitals on the molecular bridge, with a rather low coupling to the electrode states.

**Figure 4:**
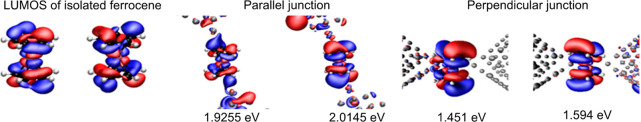
Isosurfaces of the calculated orbitals that dominate electronic transport. Left: Isosurface plots of the two (degenerate) LUMOs of an isolated ferrocene. Center and right, respectively: isosurface plots of selected electron wave functions of the Ag–ferrocene–Ag junction and their energies (with respect to Fermi energy) for the parallel and perpendicular configurations (at interelectrode separations of 9.81 and 6.2 Å, which correspond to the blue curves in Figure 3a,b). These energies lie in the immediate vicinity of the unoccupied transmission resonances. All isosurfaces contain 93% of the wave function. The plots also contain ball-and-stick models of the structures (color coding of the atoms: white (H), black (C), pink (Fe), and silver (Ag)).

Note that both Au and Ag form molecular junctions with metallocenes, and due to the dominant role of frontier s states for both metals, a similar behavior for Au–ferrocene–Au junctions may be expected. In ultrahigh vacuum conditions (including cryogenic vacuum conditions), the elongation of Au junctions produces atomic chains that may complicate data analysis. Therefore, in this study, we preferred to use Ag electrodes. Finally, we note that our calculations do not take into consideration the effect of electrode polarization.^26,27^ However, the good agreement between our experiments and calculations indicates that this effect is not dominant in the examined case.

To conclude, we showed by experiments and calculations that mechanical gating of molecular junctions depends on the orientation of the molecule in the junction. In the extreme demonstrated case, the same molecular junction can either experience mechanical gating or not, depending on the molecular orientation with respect to the electrodes, as well as on the nature of the interaction between the molecular orbitals and the continuum states of the electrodes. These findings emphasize the importance of geometry and local orbital structure in the context of charge transfer across metal-molecule interfaces and point toward a way to control mechanical gating of charge, spin, and heat transport in molecular junctions.

## Supporting Information Available

Experimental methods, additional experimental data including gating reversibility, calculation methods, differential conductance interpretation, and gating mechanism analysis (PDF)

## References

[ref1] Smit R. H. M.; Noat Y.; Untiedt C.; Lang N. D.; Van Hemert M. C.; Van Ruitenbeek J. M. Measurement of the Conductance of a Hydrogen Molecule. Nature 2002, 419 (6910), 906–909. 10.1038/nature01103. 12410305

[ref2] Stipe B. C.; Rezaei M. A.; Ho W. Single-Molecule Vibrational Spectroscopy and Microscopy. Science (80-) 1998, 280 (5370), 1732–1735. 10.1126/science.280.5370.1732. 9624046

[ref3] Park H.; Park J.; Lim A. K. L.; Anderson E. H.; Alivisatos A. P.; McEuen P. L. Nanomechanical Oscillationsinasingle-C60transistor. Nature 2000, 407, 57 10.1038/35024031. 10993069

[ref4] Hihath J.; Arroyo C. R.; Rubio-Bollinger G.; Tao N.; Agraït N. Study of Electron-Phonon Interactions in a Single Molecule Covalently Connected to Two Electrodes. Nano Lett. 2008, 8 (6), 1673–1678. 10.1021/nl080580e. 18457456

[ref5] Rakhmilevitch D.; Korytár R.; Bagrets A.; Evers F.; Tal O. Electron-Vibration Interaction in the Presence of a Switchable Kondo Resonance Realized in a Molecular Junction. Phys. Rev. Lett. 2014, 113 (23), 1–6. 10.1103/PhysRevLett.113.236603. 25526145

[ref6] Tal O.; Krieger M.; Leerink B.; Van Ruitenbeek J. M. Electron-Vibration Interaction in Single-Molecule Junctions: From Contact to Tunneling Regimes. Phys. Rev. Lett. 2008, 100 (19), 1–4. 10.1103/PhysRevLett.100.196804. 18518474

[ref7] Stefani D.; Weiland K. J.; Skripnik M.; Hsu C.; Perrin M. L.; Mayor M.; Pauly F.; Van Der Zant H. S. J. Large Conductance Variations in a Mechanosensitive Single-Molecule Junction. Nano Lett. 2018, 18 (9), 5981–5988. 10.1021/acs.nanolett.8b02810. 30134105 PMC6143316

[ref8] Camarasa-Gómez M.; Hernangómez-Pérez D.; Inkpen M. S.; Lovat G.; Fung E. D.; Roy X.; Venkataraman L.; Evers F. Mechanically Tunable Quantum Interference in Ferrocene-Based Single-Molecule Junctions. Nano Lett. 2020, 20 (9), 6381–6386. 10.1021/acs.nanolett.0c01956. 32787164

[ref9] Perrin M. L.; Verzijl C. J. O.; Martin C. A.; Shaikh A. J.; Eelkema R.; Van Esch J. H.; Van Ruitenbeek J. M.; Thijssen J. M.; Van Der Zant H. S. J.; Dulić D. Large Tunable Image-Charge Effects in Single-Molecule Junctions. Nat. Nanotechnol 2013, 8 (4), 282–287. 10.1038/nnano.2013.26. 23503093

[ref10] Pump F.; Temirov R.; Neucheva O.; Soubatch S.; Tautz S.; Rohlfing M.; Cuniberti G. Quantum Transport through STM-Lifted Single PTCDA Molecules. Appl. Phys. A Mater. Sci. Process 2008, 93 (2), 335–343. 10.1007/s00339-008-4837-z.

[ref11] Quek S. Y.; Kamenetska M.; Steigerwald M. L.; Choi H. J.; Louie S. G.; Hybertsen M. S.; Neaton J. B.; Venkataraman L. Mechanically Controlled Binary Conductance Switching of a Single-Molecule Junction. Nat. Nanotechnol 2009, 4 (4), 230–234. 10.1038/nnano.2009.10. 19350032

[ref12] Scott G. D.; Natelson D. Kondo Resonances in Molecular Devices. ACS Nano 2010, 4 (7), 3560–3579. 10.1021/nn100793s. 20568709

[ref13] Toher C.; Temirov R.; Greuling A.; Pump F.; Kaczmarski M.; Cuniberti G.; Rohlfing M.; Tautz F. S. Electrical Transport through a Mechanically Gated Molecular Wire. Phys. Rev. B - Condens Matter Mater. Phys. 2011, 83 (15), 1–12. 10.1103/PhysRevB.83.155402.

[ref14] Rincón-García L.; Ismael A. K.; Evangeli C.; Grace I.; Rubio-Bollinger G.; Porfyrakis K.; Agraït N.; Lambert C. J. Molecular Design and Control of Fullerene-Based Bi-Thermoelectric Materials. Nat. Mater. 2016, 15 (3), 289–293. 10.1038/nmat4487. 26641017

[ref15] Walkey M. C.; Peiris C. R.; Ciampi S.; Aragonès A. C.; Domínguez-Espíndola R. B.; Jago D.; Pulbrook T.; Skelton B. W.; Sobolev A. N.; Díez Pérez I.; Piggott M. J.; Koutsantonis G. A.; Darwish N. Chemically and Mechanically Controlled Single-Molecule Switches Using Spiropyrans. ACS Appl. Mater. Interfaces 2019, 11 (40), 36886–36894. 10.1021/acsami.9b11044. 31522492

[ref16] Beebe J. M.; Kim B.; Gadzuk J. W.; Frisbie C. D.; Kushmerick J. G. Transition from Direct Tunneling to Field Emission in Metal-Molecule-Metal Junctions. Phys. Rev. Lett. 2006, 97 (2), 026801 10.1103/PhysRevLett.97.026801. 16907471

[ref17] Beebe J. M.; Kim B.; Frisbie C. D.; Kushmerick J. G. Measuring Relative Barrier Heights in Molecular Electronic Junctions with Transition Voltage Spectroscopy. ACS Nano 2008, 2 (5), 827–832. 10.1021/nn700424u. 19206478

[ref18] Huisman E. H.; Guédon C. M.; Van Wees B. J.; Van Der Molen S. J. Interpretation of Transition Voltage Spectroscopy. Nano Lett. 2009, 9 (11), 3909–3913. 10.1021/nl9021094. 19685928

[ref19] Trouwborst M. L.; Martin C. A.; Smit R. H. M.; Guédon C. M.; Baart T. A.; Van Der Molen S. J.; Van Ruitenbeek J. M. Transition Voltage Spectroscopy and the Nature of Vacuum Tunneling. Nano Lett. 2011, 11 (2), 614–617. 10.1021/nl103699t. 21214259

[ref20] Vilan A.; Cahen D.; Kraisler E. Rethinking Transition Voltage Spectroscopy within a Generic Taylor Expansion View. ACS Nano 2013, 7 (1), 695–706. 10.1021/nn3049686. 23236949

[ref21] Song H.; Kim Y.; Jang Y. H.; Jeong H.; Reed M. A.; Lee T. Observation of Molecular Orbital Gating. Nature 2009, 462 (7276), 1039–1043. 10.1038/nature08639. 20033044

[ref22] Bruot C.; Hihath J.; Tao N. Mechanically Controlled Molecular Orbital Alignment in Single Molecule Junctions. Nat. Nanotechnol 2012, 7 (1), 35–40. 10.1038/nnano.2011.212. 22138861

[ref23] Pal A. N.; Li D.; Sarkar S.; Chakrabarti S.; Vilan A.; Kronik L.; Smogunov A.; Tal O. Nonmagnetic Single-Molecule Spin-Filter Based on Quantum Interference. Nat. Commun. 2019, 10 (1), 5565 10.1038/s41467-019-13537-z. 31804498 PMC6895237

[ref24] Evers F.; Korytár R.; Tewari S.; Van Ruitenbeek J. M. Advances and Challenges in Single-Molecule Electron Transport. Rev. Mod. Phys. 2020, 92 (3), 35001 10.1103/RevModPhys.92.035001.

[ref25] Schreckenbach G. The 57Fe Nuclear Magnetic Resonance Shielding in Ferrocene Revisited. A Density-Functional Study of Orbital Energies, Shielding Mechanisms, and the Influence of the Exchange-Correlation Functional. J. Chem. Phys. 1999, 110 (24), 11936–11949. 10.1063/1.479133.

[ref26] Repp J.; Meyer G.; Stojković S. M.; Gourdon A.; Joachim C. Molecules on Insulating Films: Scanning-Tunneling Microscopy Imaging of Individual Molecular Orbitals. Phys. Rev. Lett. 2005, 94 (2), 1–4. 10.1103/PhysRevLett.94.026803. 15698209

[ref27] Neaton J. B.; Hybertsen M. S.; Louie S. G. Renormalization of Molecular Electronic Levels at Metal-Molecule Interfaces. Phys. Rev. Lett. 2006, 97 (21), 1–4. 10.1103/PhysRevLett.97.216405. 17155759

