# Supplementary material for: Structural Regulation of Mechanical Gating in Molecular Junctions
Source: Nano Lett. 2023 May 2;23(9):3775–80. doi: 10.1021/acs.nanolett.3c00043 (PMC10176572; doi:10.1021/acs.nanolett.3c00043)
Supplement: Supplementary file 1 — nl3c00043_si_001.pdf [file nl3c00043_si_001.pdf]

Supporting Information

Structural regulation of mechanical gating in molecular junctions

Biswajit Pabi[1], Jakub Šebesta[2,3], Richard Korytár[2], Oren Tal[4], and Atindra Nath Pal[1,4]

1. Department of Condensed Matter and Materials Physics, S. N. Bose National Centre for Basic Sciences, Sector III, Block JD, Salt Lake, Kolkata700106, India

2. Department of Condensed Matter Physics, Faculty of Mathematics and Physics, Charles University, CZ-121 16 Praha 2, Czech Republic

3. Materials Theory, Department of Physics and Astronomy, Uppsala University Box 516, 751 20 Uppsala, Sweden

4. Department of Chemical and Biological Physics, Weizmann Institute of Science, Rehovot 7610001, Israel

Content:

1. Experimental methods and conductance characterization of Ag and Ag-ferrocene junction.

2. Additional experimental data.

3. Reversible Mechanical gating.

4. Ab initio calculations of structure and electronic transport.

5. Interpretation of differential conductance curves in break junction experiments.

6. DFT analysis of the physical mechanism driving the gating effect.

1. Experimental methods and conductance characterization of Ag and Ag-ferrocene junction.

A mechanically controllable break junction set up is used to form molecular junction. A Ag wire (99.997%, 0.1 mm, Alfa Aesar) with a notch at its middle is fixed on top of a flexible substrate (1mm thick phosphor bonze covered by 100µm Kapton foil). This structure is placed in a vacuum chamber that is cooled by liquid helium to ~ 4.2K. Using a three-point bending mechanism, the substrate is pushed and bent. This process stretches the wire notch, resulting a gradual reduction in its cross section down to the atomic scale. Fine bending of the substrate is achieved by a piezo electric actuator (PI P- 882 PICMA) which is driven by a 24-bit DAQ card (PCI 4461 - National Instruments), followed by a piezo driver (SVR 150/1, Piezomechanik). Sufficient bending of the substrate results wire breaking into two wire segments with freshly exposed atomic tips that are formed in an ultraclean cryogenic environment. These sharp wire segments serve as electrodes. Molecular junctions are prepared by continuously breaking and reforming a metallic atomic contact between the electrode tips, while sublimating ferrocene molecules (99.5%, Alpha Aesar, further purified in situ) from a locally heated molecular source towards the atomic contact. Direct current (d.c.) conductance is measured when the junction is gradually pulled apart to form conductance versus distance traces. A constant 500 mV from the DAQ card is supplied to a divider by 10 (to improve signal to noise ratio) and the resulted 50 mv bias is applied across the junction. The resulted current output from the junction is amplified by a I/V preamplifier (Femto DLPCA-200), and the amplified signal is recorded by the same DAQ card. The d.c. conductance of the junction is thus the measured current divided by the applied voltage (50 mV). Inter-electrode displacement is estimated based on the dependence of the tunneling current on the electrode separation, following a standard process[1]. Differential conductance spectra (dI/dV versus V) is recorded using a standard lock-in technique. A reference sine signal from a lock-in amplifier (SR830) with a peak to peak voltage of 10 mv and a frequency of ~3.333 kHz is added to a d.c. voltage and the total voltage is divided by 10 to improve signal to noise ratio. The response alternating current (a.c.) is probed by a lock-in amplifier (SR830) and recorded by the DAQ card The differential conductance spectra are obtained by dividing the alternating current signal (dI) with the applied alternating voltage bias (dV), as a function of a swiped d.c. voltage bias (V).

The d.c. conductance of the junction (current/voltage) is recorded during repeated junction stretching as a function of relative interelectrode displacement. First, a bare Ag junction was characterized. Figure S1a presents in blue examples for conductance traces as a function of interelectrode displacement. During the elongation of the Ag junction, the conductance decreases in steps when the contact diameter is reduced. The last conductance plateau at ~1 G_0 ($G_0 = 2e^2/h$, is the conductance quantum, where e is the electron charge and h is the Plank's constant) provides the conductance of a single atom contact between the Ag

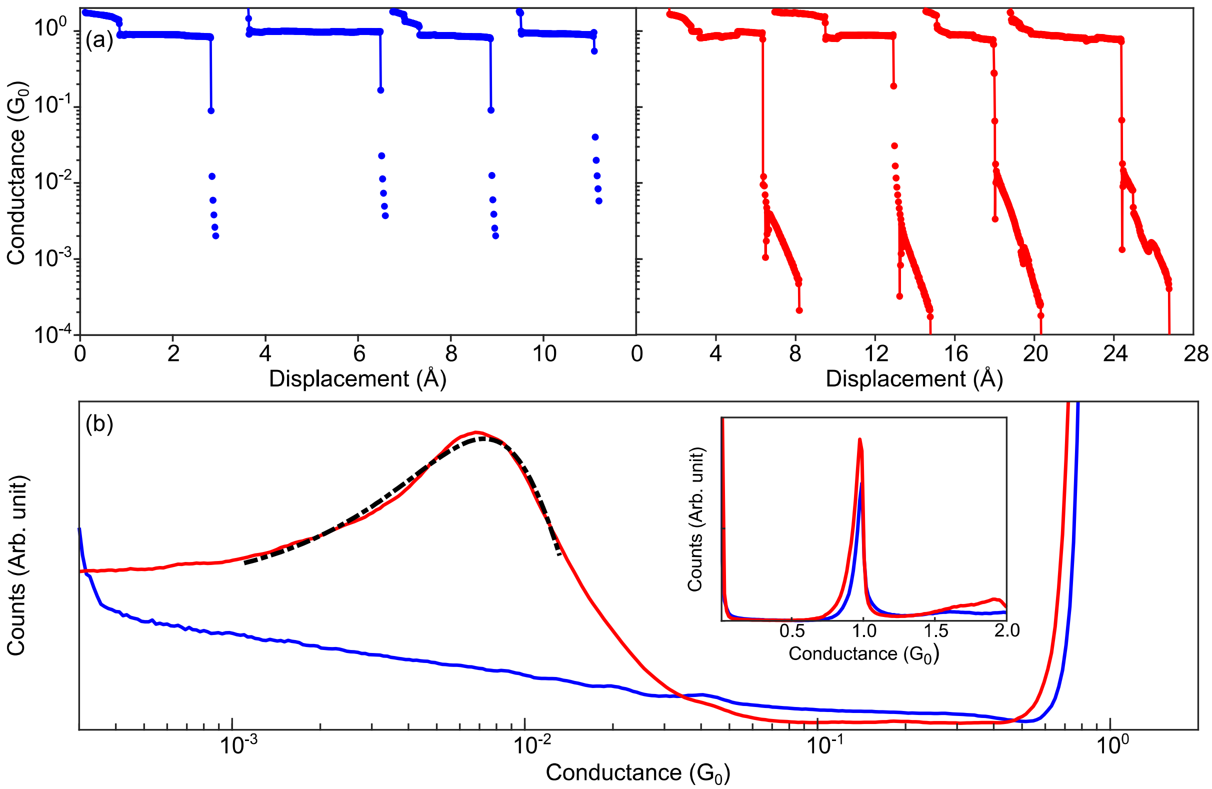

Figure S1. Conductance characterization of Ag and Ag-ferrocene junction. (a) Left and right panel: Traces of conductance vs. interelectrode displacement recorded during the breaking of Ag atomic-scale junctions before (blue) and after (red) the insertion of ferrocene molecules, recorded at 50 mV bias voltage. Traces are shifted horizontally for clarity. (b) Conductance histogram for Ag (blue) and Ag-ferrocene (red) junctions, prepared from 5,000 and 10,000 consecutive conductance traces using 80 bins per decade. Black dash dot line represents a Gaussian fitting to the observed peak. Inset: Conductance histogram of the same junctions, constructed from 300 bins in a linear scale.

electrode tips[2]. After the insertion of ferrocene molecules, tilted plateaus below the ~1 G_0 step are clearly seen (Figure S1a, red traces). To statistically characterize the most probable conductance features, conductance histograms (Figure S1b) are constructed from 5,000 and 10,000 consecutive conductance-displacement traces for Ag junctions before (blue) and after (red) the introduction of ferrocene, respectively. While the conductance histogram for bare Ag junctions reveals a peak at ~1 G_0 (Inset of Figure S1b) that is associated with the most probable conductance of single Ag atom contacts, after the introduction of ferrocene an additional conductance peak beneath the 1G_0 peak is observed. Gaussian fitting of the corresponding peak, shown by a black dash dot line in Figure S1b yields a most probable conductance value of $(7.25 \pm 0.06) \times 10^{-3}$ G_0 for Ag-ferrocene molecular junctions. The error corresponds to 2SD or ±2 standard deviations of the fitted Gaussian.

2. Additional experimental data.

Each set contains data measured for a single junction at different interelectrode distances.

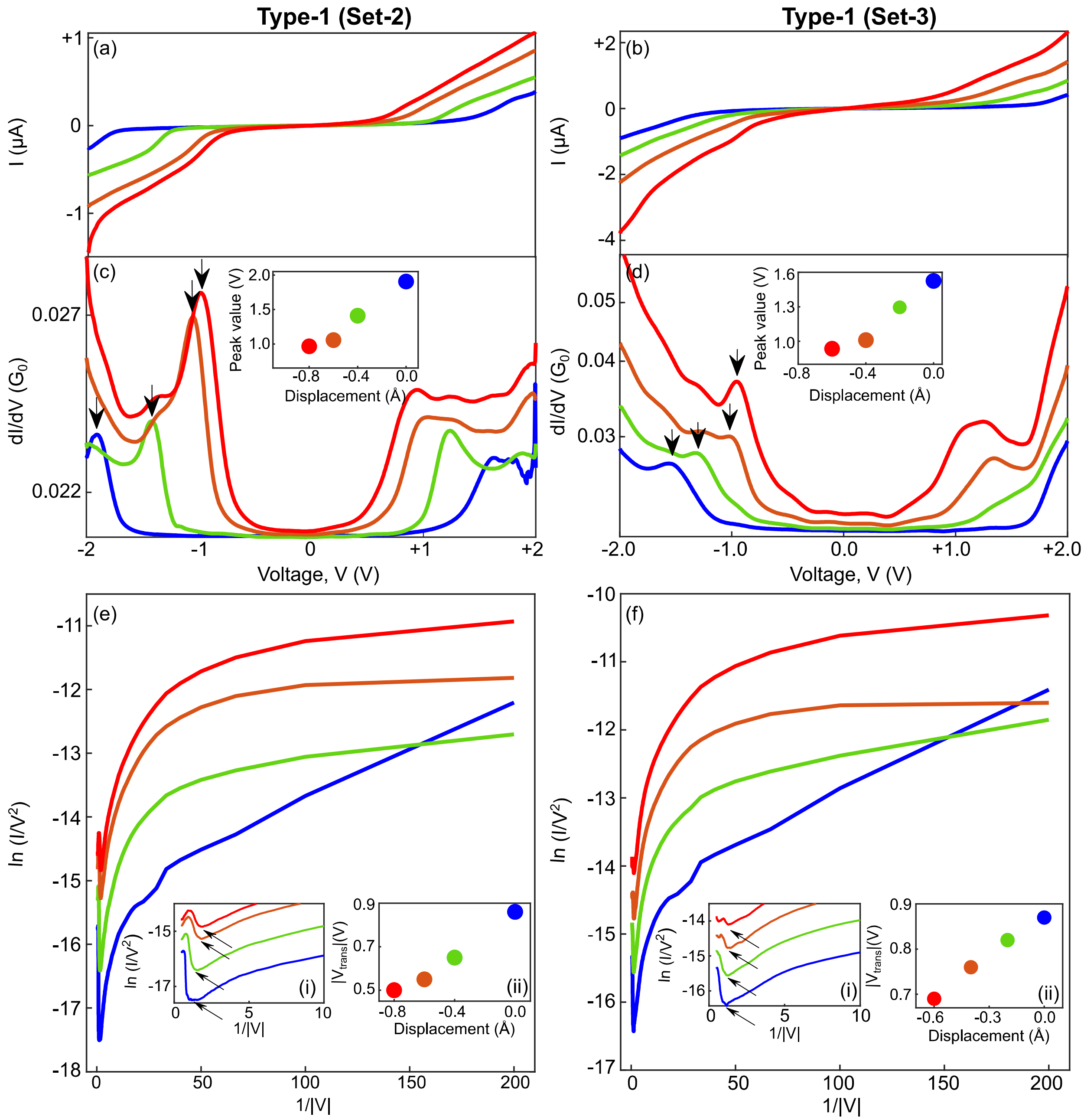

Figure S2. Current-voltage curves, differential conductance spectra, and TVS plots for type 1. (a,b) Current vs. voltage measured at different interelectrode displacements in Ag-ferrocene junctions with mechanical gating response (type 1). (c) Differential conductance vs. voltage for the junction studied in a. (d) Same as (c) but with data collected for the molecular junction studied in b. Insets (c,d) Absolute values of peak position (marked with arrows in c,d) vs. interelectrode displacement. (e-f) TVS plots constructed from the same I-V spectra presented in a,b. For consistency, the negative side of the I-V curves is considered for TVS analysis. Insets (i): Zoomed view of the TVS plots. Insets (ii): Transition voltage (absolute values) vs. interelectrode displacement.

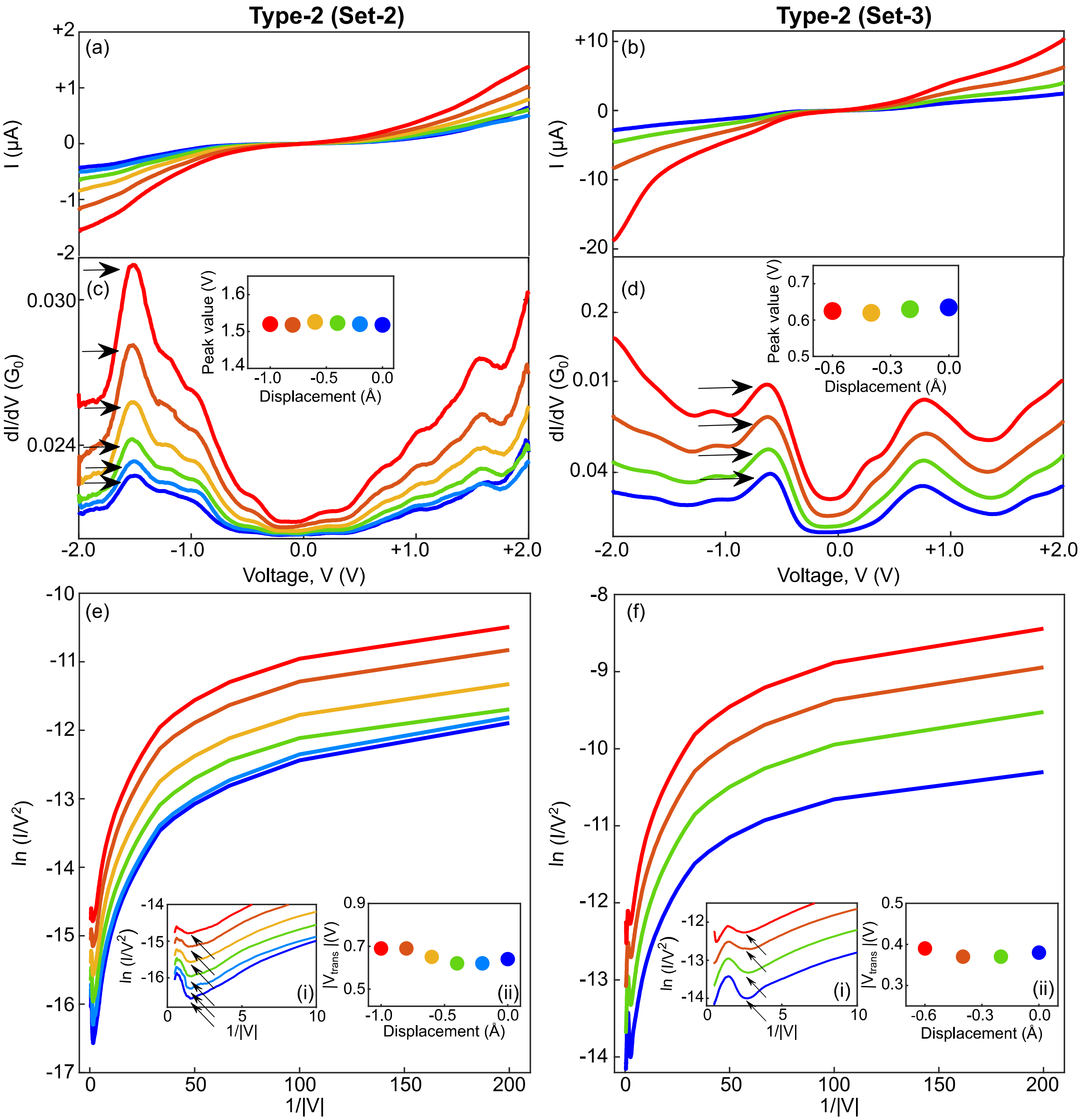

Figure S3. Current-voltage curves, differential conductance spectra, and TVS plots for type 2. (a,b) Current vs. voltage measured at different interelectrode displacements in Ag-ferrocene junctions with no mechanical gating response (type 2). (c) Differential conductance vs. voltage for the junction studied in a. (d) Same as (c) but with data collected for the molecular junction studied in b. Insets (c,d) Absolute values of peak position (marked with arrows in c,d) vs. interelectrode displacement. (e-f) TVS plots constructed from the same I-V spectra presented in a,b. Insets (i): Zoomed view of the TVS plots. Insets (ii): Transition voltage (absolute values) vs. interelectrode displacement.

In view of the calculations in Figure 3, main text, the differential conductance is expected to be similar for parallel and perpendicular ferrocene junctions in a wide range around zero bias voltage. Figure 2 in the main text and Figures S2 and S3 in the Supplementary Information reveal that the measured differential conductance of types 1 and 2 are similar as well. We therefore do not distinguish between the two configurations based on the conductance of the junction. The molecular junctions are first prepared, and the identification is done a posteriori by repeated dI/dV vs. V measurements for different interelectrode separations.

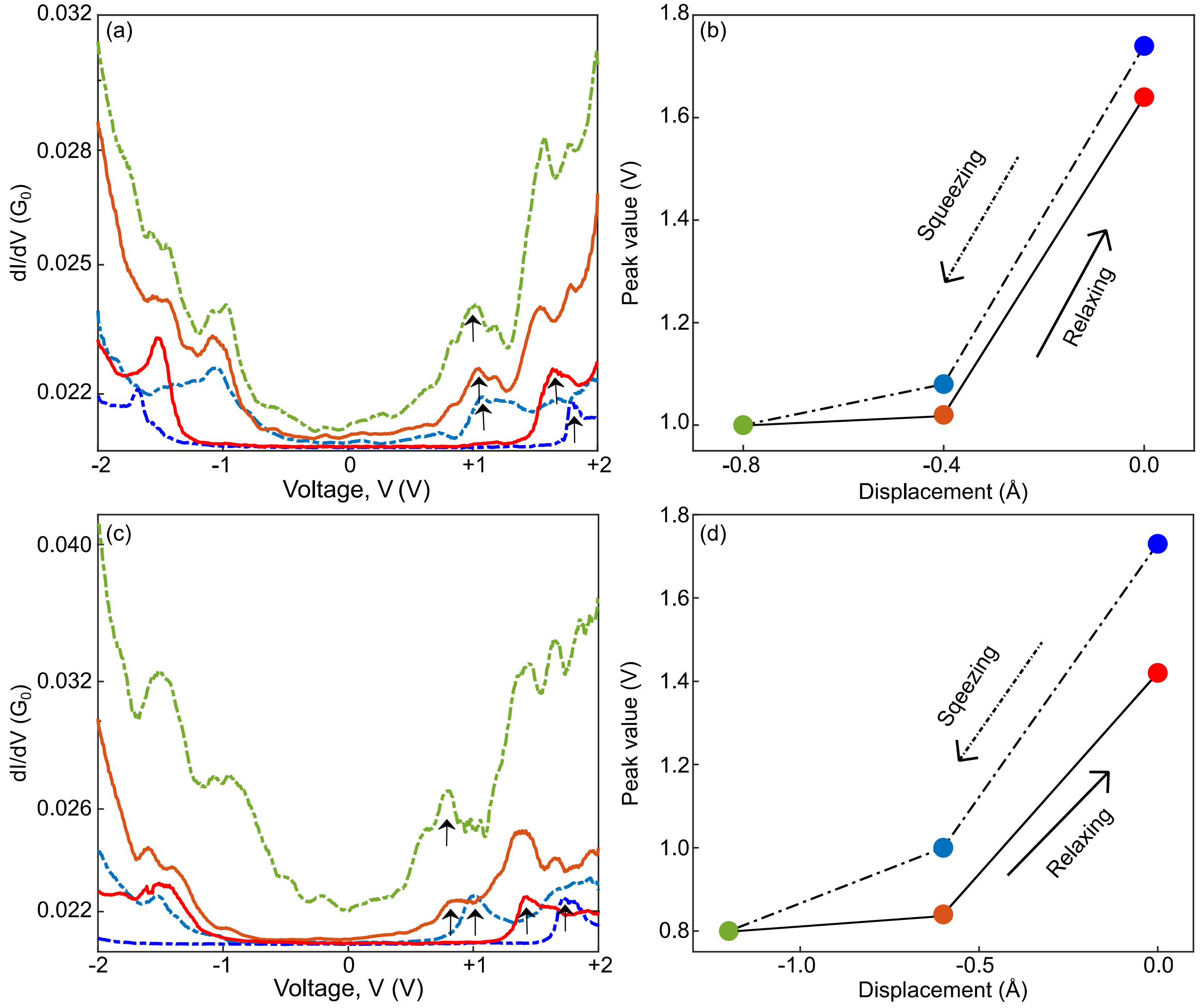

Figure S4. Differential conductance spectra and evolution of its peak position with mechanical manipulation. (a) Differential conductance as a function of applied voltage of the Ag-ferrocene junction where spectra corresponding to squeezing (relaxing) is plotted by the dash-dotted (solid). Peak position of each spectra is denoted by arrow and interelectrode separation corresponding to each color is shown in b. (b) Absolute values of the peak position as a function of interelectrode displacement where squeezing (relaxing) process is represented by dash-dotted (solid) line (c, d) similar analysis like (a, b) for another junction realization.

Note that the molecular junctions are prepared by breaking an atomic contact between the electrodes in the presence of adsorbed molecules or by bringing together the electrode tips of an already broken junction to arbitrarily capture a molecule between the two electrodes. In both cases, we do not observe a preference for the realization of type 1 or 2. We therefore arbitrarily obtain both types.

3. Reversible Mechanical gating.

Figures S4a and S4c provide examples for the extent of mechanical gating reversibility. The first three spectra (dashed curves in blue, light-blue, and green) are recorded for sequential reduction in the interelectrode separation, and next two spectra (solid curves in orange and red) are obtained for sequential increase in the interelectrode separation up to the primary separation. The gating reversibility is clearer in Figures S4b and S4d that present the voltage value of the arrowed peaks as a function of interelectrode separation. For both cases, the peaks are shifted to lower (higher) voltage during junction squeezing (elongation). The values during squeezing and elongation are rather similar but not identical, possibly due to molecular junction rearrangements.

4. Ab initio calculations of structure and electronic transport.

Theoretical transmission functions were obtained by ab-initio transport calculations based on the non-equilibrium Green's function (NEGF) technique and effective scattering states given by the Kohn-Sham density functional theory (DFT), see Ref. 3 for a comprehensive overview. Utilizing the TURBOMOLE package[4,5], electronic wave-functions were described through Gaussian type orbitals of the def2-TZVP basis set[6,7], while the exchange-correlation functional of the Perdew-Burke-Ernzerhof (PBE) type was employed[8].

Structures and structural relaxation

Prior the transport calculation, possible molecule's binding conformations to the electrodes were studied through molecule's structure optimization by the quasi-Newton-Raphson method[9]. All the examined structures were non-periodic clusters composed of the ferrocene molecule and an attached pair of Ag electrodes with a pyramidal shape, as schematically depicted in the insets of Figure 2 and in Figure S5.

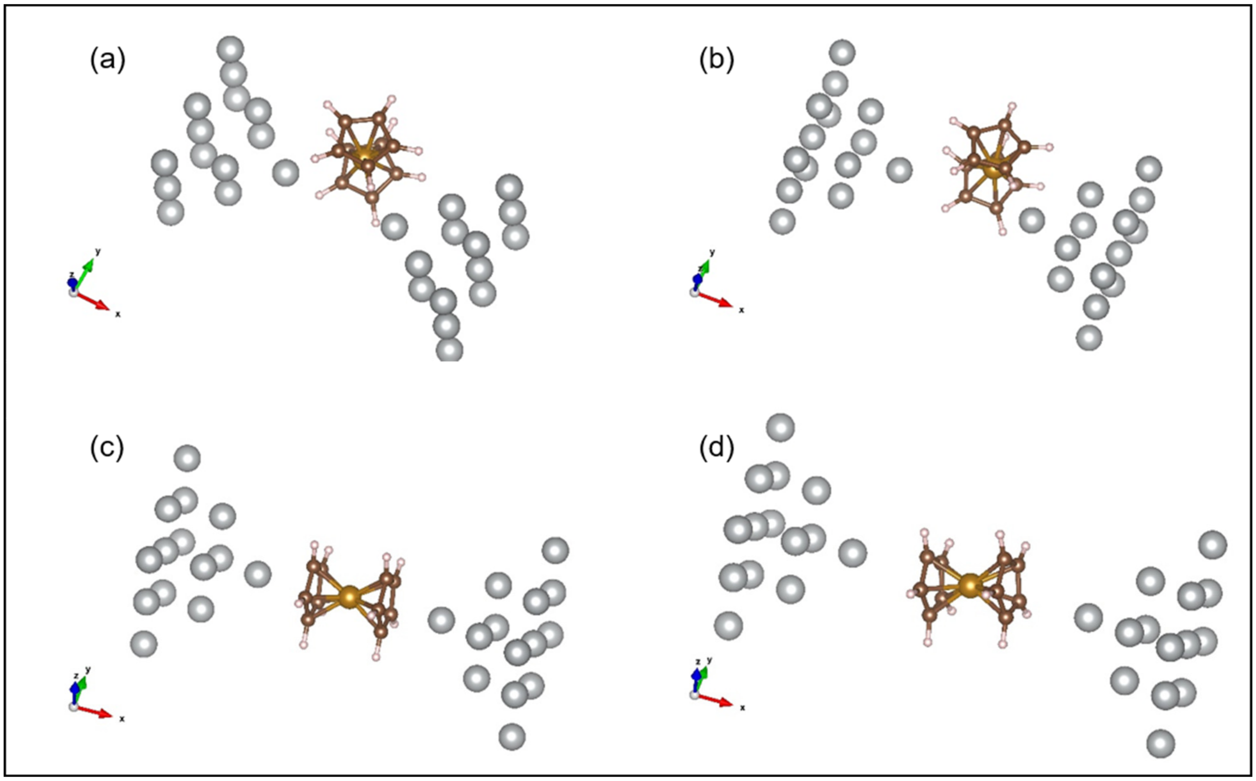

Figure S5. Relaxed structures. Perpendicular molecule orientation with respect to the electrode axis: (a) d=5.2 Å (b) d=6.2Å. Parallel molecule orientation: (c) d=8.1 Å (d) d=9.8Å.

The Ag electrodes were cut out of a face-centered Ag crystal along the (100) direction considering a bulk lattice parameter[10]. They consist of an apex atom and a set of atomic layers with a growing number of atoms. During relaxation, we kept the Ag atomic positions fixed. The sequence of pyramids was necessary to study of the conductance's convergence when the size of the pyramidal electrode tips increases. Due to computational demands, the structure optimization was performed for clusters with three-layered leads and the molecule coordinates were pre-relaxed in the gas phase.

Quantum transport

Differential conductance was calculated via NEGF formalism[3] employing the AITRANSS package[11–13]. The infinite electrodes were accounted for by self-energy operators applied to the Ag pyramids following Ref. 13. At a given voltage V, the electric current is given by the expression

$$I(V) = \frac{2e}{h}\int_{-\infty}^{\infty} T(E,V)\,[f_L(E,V) - f_R(E,V)]dE \qquad \text{(S1)}$$

where the factor 2 accounts for spin degeneracy, $T(E,V)$ is the transmission function and $f_{L,R}$ denotes the Fermi-Dirac distributions of the left (L) and right (R) electrodes. We assumed that the external bias voltage

causes a symmetric shift of the left and right chemical potentials, $\mu_{LR} = \mu \pm \frac{eV}{2}$, where μ stands for the equilibrium chemical potential. Further, we adopted the zero-temperature form of $f_{L,R}$; this approximation is valid as long the transmission function $T(E,V)$ varies slowly within the energy range ∈ $\left(\mu_{L,R} - k_B T, \mu_{L,R} + k_B T\right)$, where $k_B T$ is the working temperature multiplied by the Boltzmann constant. Considering the expression $f_{L,R}(E,V) = \theta\left(\mu \pm \frac{eV}{2} - E\right)$, it follows that the current and its derivative are given by

$$I(V) = \frac{2e}{h}\int_{E_F-\frac{eV}{2}}^{E_F+\frac{eV}{2}} T(E,V)\, dE \qquad \text{(S2)}$$

$$\frac{dI}{dV} = \frac{2e^2}{h}\frac{1}{2}\left[T\left(E_F - \frac{eV}{2}\right) + T\left(E_F + \frac{eV}{2}\right)\right] \qquad \text{(S3)}$$

where the Fermi energy E_F equals the chemical potential at T = 0. We neglected the explicit voltage dependence in the transmission, which can quantitatively, and sometimes qualitatively change the differential conductance. The bias voltage can lead to non-equilibrium Stark effect of the resonances[11]. This effect can simply be understood as orbital modifications due to the presence of an effective electric field induced by the voltage V. Consequently, the coupling of an orbital to the two electrodes is more asymmetric at $V \neq 0$ than at V = 0 and the heights of the corresponding transmission peaks diminish. Therefore, the theoretical differential conductance of type-1 conformation could overemphasize the resonance heights and the inclusion of non-equilibrium Stark effect would diminish them, making them more consistent with the experimental data.

5. Interpretation of differential conductance curves in break junction experiments.

In the examined molecular junctions, the two resonances seen in the differential conductance curves (dI/dV vs. V) above and below zero voltage (see Figure 2c, Figure S2c,d, and the wide peaks in Figure 3c) shift towards zero voltage in response to junction squeezing. However, as explained below, we conclude that both HOMO and LUMO are shifted downwards together (*i.e.,* gating effect) with no significant changes in the HOMO - LUMO gap, as illustrated in Figure 3a.

The interpretation of differential conductance curves can be fairly different for scanning tunneling microscope (STM) and break junction experiments, due to the very asymmetric electrode-molecule coupling in a typical STM measurement and a rather symmetric coupling in mechanically controllable break junctions. Focusing first on our break junction experiments, in the ideal case of an identical electrode-molecule coupling for both electrodes, an applied voltage across the junction is distributed evenly on the electrode-molecule interfaces as schematically illustrated in Figure S6a

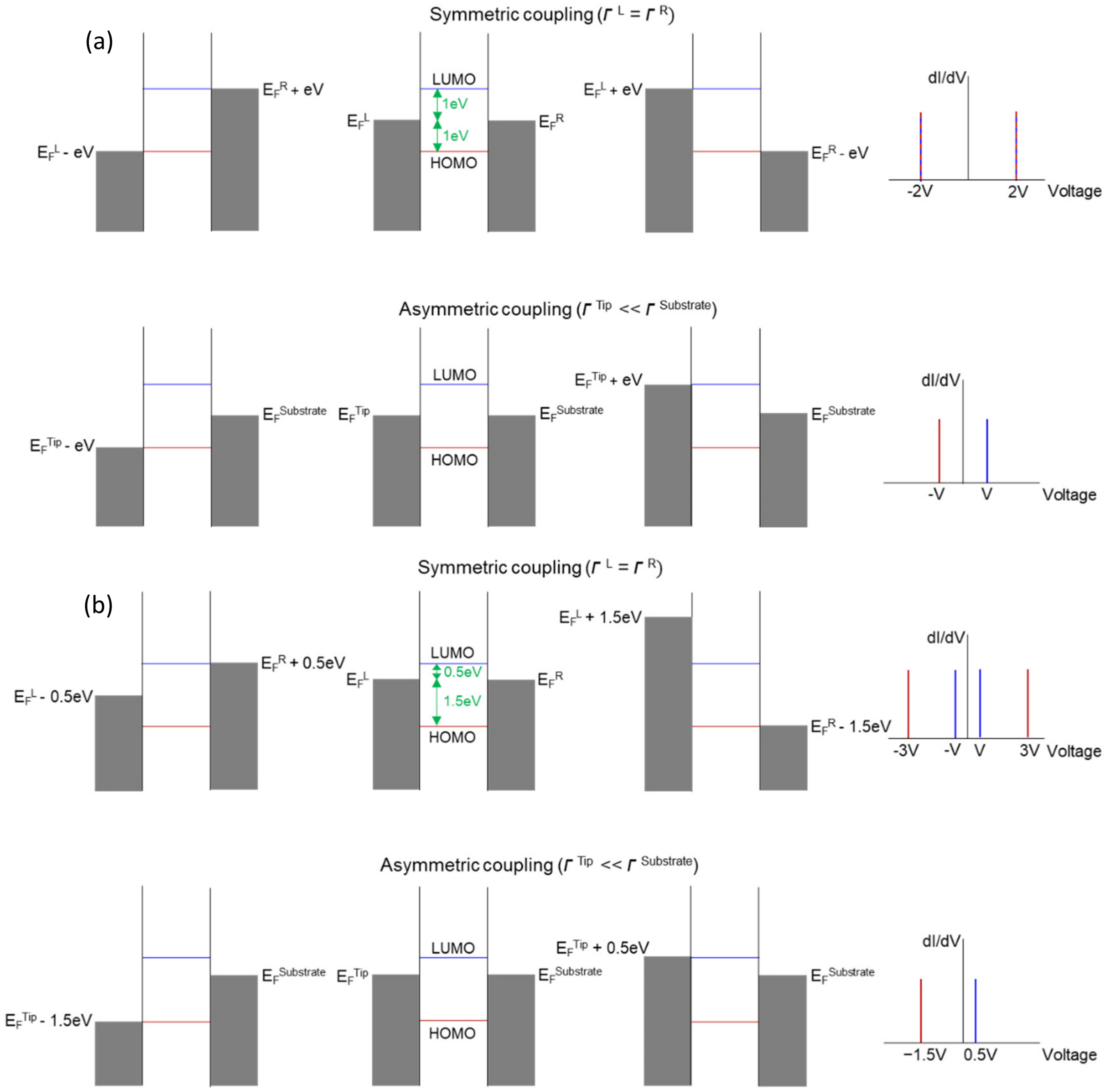

Figure S6. (a) Top Panel: Cartoon of energy scheme and differential conductance curve for symmetric electrode-molecule coupling ($\Gamma^R = \Gamma^L$; Γ^i – right and left ($i = R, L$) electrode-molecule coupling). The HOMO (red) and LUMO (blue) are located 1eV below and above the Fermi energy. Since the voltage drops equally across the junction, when a voltage of $\pm$2V is applied to the junction the quasi Fermi levels of the electrodes are aligned with both HOMO and LUMO, at -eV and eV. As a result, both HOMO and LUMO contribute to the resonance peaks seen in the differential conductance at $\pm$eV. Bottom Panel: Cartoon of energy scheme and differential conductance curve for asymmetric electrode-molecule coupling as in traditional STM experiments (molecule coupling to the tip is much smaller that its coupling to the substrate, $\Gamma^{Tip} \ll \Gamma^{Substrate}$). Also, here the HOMO (red) and LUMO (blue) are located $\pm$ eV below and a above the Fermi energy. When a voltage of $\pm$V is applied to the junction the quasi Fermi level of the tip is aligned with either the HOMO or LUMO, since the voltage drops on the vacuum gap between the tip and the molecule. As a result, either the HOMO or LUMO contribute to the resonance peaks seen in the differential conductance at $\pm$eV. (b) Same as (a), but with HOMO and LUMO located 1.5eV below and 0.5eV above the Fermi energy, respectively. The voltage range required to probe the levels varies accordingly.

top panel (we neglect here a possible voltage drop on the molecule for simplicity). For a large enough voltage, both HOMO and LUMO are sampled at the same energy window, as illustrated in the left and right energy schemes in Figure S6a. In case of a similar HOMO and LUMO distance from the Fermi energy, the dI/dV curve shows a resonance in the differential conductance curve with a shape that is an outcome of both HOMO and LUMO shapes. In Figure S6, we do not illustrate the resonance shapes but the orange curve in Figure 3c, provides a calculated example (sharp HOMO peaks on top of a wide LUMO peak). The observed resonance appears at a voltage that is given by the energy of the HOMO and LUMO multiplied by 2. This factor is a consequence of the voltage division between the two metal-molecule interfaces, where the alignment of the quasi Fermi energy with either the HOMO or LUMO takes place at an applied voltage (times *e*) equal to twice the level's energy: Ve=2E. Note that a similar scaling is observed in Figures 3a and 3c, and the description here is given mathematically by Eq. (3) in Supporting Information Section 4.

In the opposite limit, where the electrode-molecule coupling of the two electrodes is highly asymmetric as in traditional STM experiments, the situation is very different as illustrated in Figure S6 bottom panel. Here, an applied voltage across the junction falls mainly on the insulating gap between the electrode tip and the molecule (in Figure S6, we assume that all the voltage drops on the tip-molecule interface for simplicity). As described in the figure, depending on the voltage sigh either the HOMO or LUMO are probed in the dI/dV curve separately, leading to a resonance in the differential conductance curve located at a voltage corresponding (in eV) to the HOMO or LUMO energy with a shape that is the outcome of the shape of each energy level individually.

We will now examine an energy level shift and how it is reflected in dI/dV curves for symmetric coupling between the electrode and molecule. As seen in Figure 3a, a shift of both HOMO and LUMO to a lower energy (i.e., a gating effect with no significant change in the HOMO-LUMO gap), is translated in the dI/dV curves in Figure 3c to a shift of the LUMO wide resonance towards zero voltage and a shift of the HOMO narrow resonance to a higher voltage in an absolute value. The dI/dV response to the HOMO and LUMO shift to a lower energy is schematically illustrated in Figure S6b for a symmetric (top) and asymmetric (bottom) coupling. In the actual measurements (Figure 2a), we do not clearly probe the very sharp resonances but the wide dI/dV resonance reveals the expected downwards shift and peak widening seen in the calculations (Figure 3c) in response to junction squeezing.

6. DFT analysis of the physical mechanism driving the gating effect.

The gating mechanism observed in our DFT calculations is based on two physical principles. First, as the junction compresses, the confinement of the molecular orbitals is augmented. Second, there is a self-consistent rearrangement of the charge density in the junction. Here, we provide insights into both phenomena based on *ab-initio* calculations.

Figure S7 shows the relative change of bond lengths upon compression. At ~10Å, compression occurs between the apex and the nearest ring (on both sides). Below 9Å this distance (Apex-C, orange data) reduced and compression now takes place mainly between the rings, *i.e.* the height of the ferrocene reduces (purple data). Interestingly, in the very squeezed limit, the vertical compression of the molecule is compensated by an increase of the C-C distance, and henceforth widening of the rings.

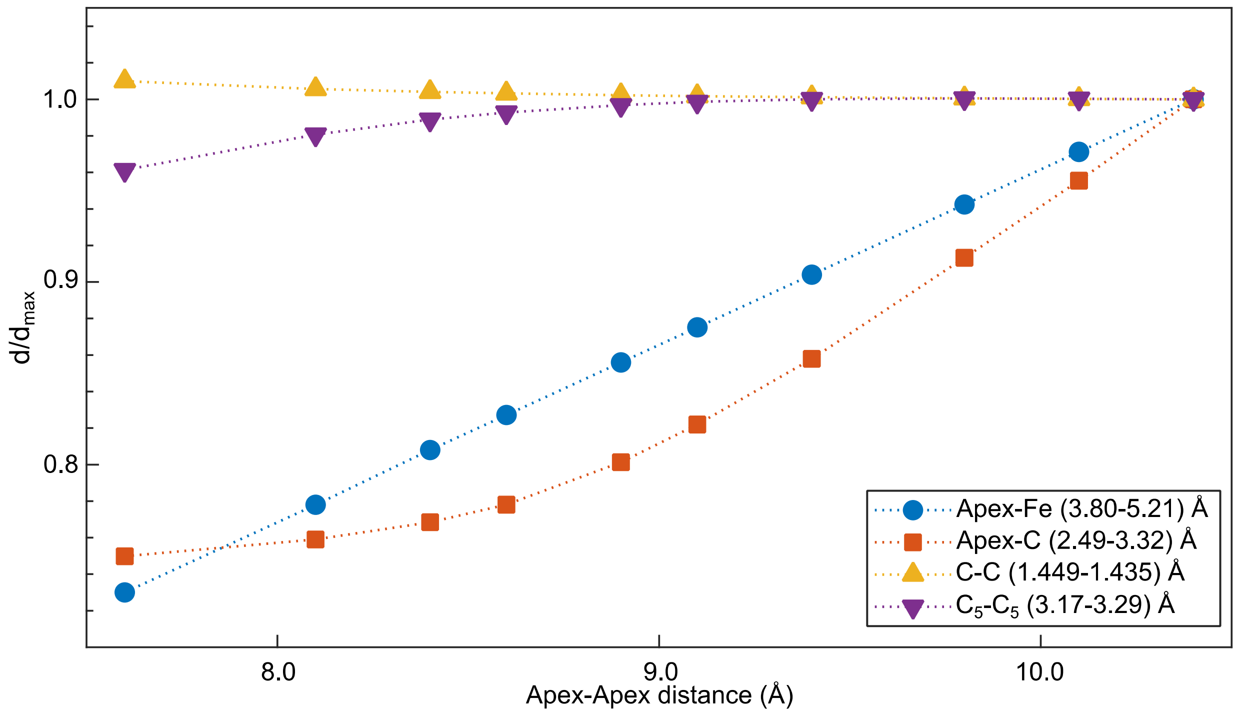

Figure S7: Relative changes of various bond lengths upon stretching, the reference values d_{max} are taken at 10.3Å. The average length of the 10 carbon bonds is denoted by C-C; the average distance of the two rings is C_5-C_5; the average distance from the apex to the 5 nearest carbons is denoted by Apex-C.

Figure S8 shows that changes in charge due to compression mainly takes place at the molecule's rings, while the charge on the iron center is almost intact. We also analyze the charge transfer from the density of states (DOS) projected onto the atomic species of the junction for two apex distances in Figures S9 a and b. Unlike the transmission function, the DOS can be understood as an energy-resolved occupation number. For both apex distances, the DOS around the Fermi energy has the form of Lorentzian-like HOMO and LUMO peaks, superimposed on a background that weakly increases with energy. The junction compression leads to a HOMO and LUMO shift downwards. Namely, change is transferred to the molecule and occupies more states. As seen in Figure S8, this excess charge is mainly accumulated on the carbon rings.

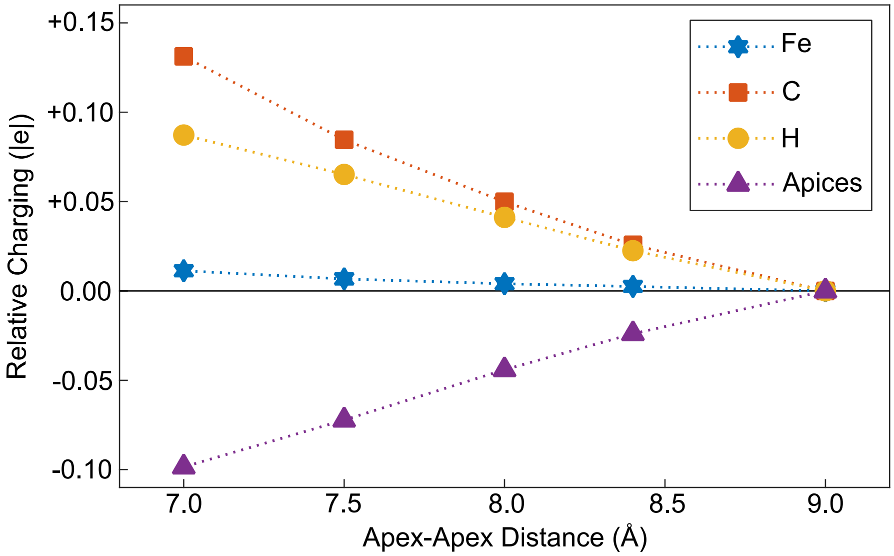

Figure S8: Change of Löwdin charges with respect to the charges at 9Å; parallel configuration.

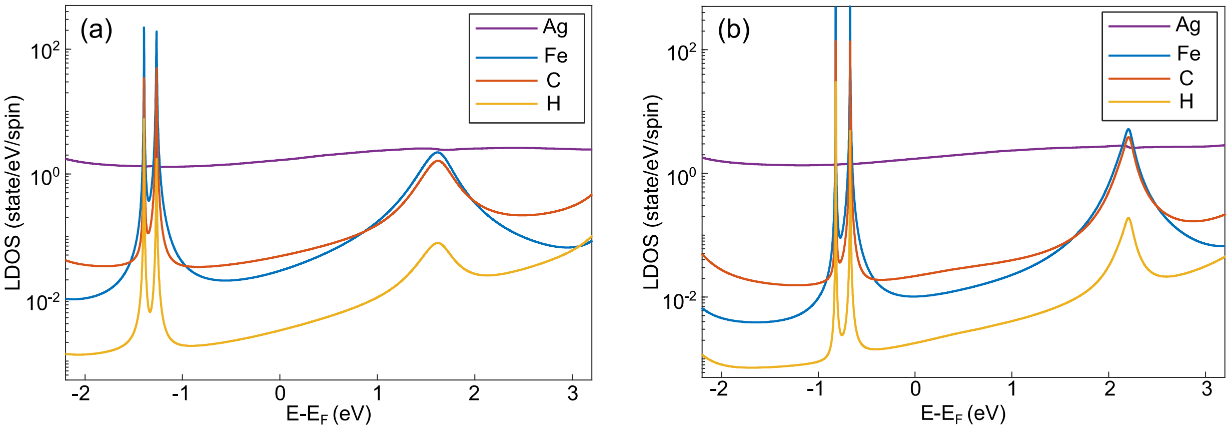

Figure S9: Density of states projected on specific elements of the parallel junction. Apex-apex distance of 7.5Å (a) and 9Å (b).

References:

(1) Untiedt, C.; Yanson, A. I.; Grande, R.; Rubio-Bollinger, G.; Agraït, N.; Vieira, S.; van Ruitenbeek, J. M. Calibration of the Length of a Chain of Single Gold Atoms. *Phys Rev B - Condens Matter Mater Phys* **2002**, *66* (8), 854181–854186. https://doi.org/10.1103/PhysRevB.66.085418.

(2) Rodrigues, V.; Bettini, J.; Rocha, A. R.; Rego, L. G. C.; Ugarte, D. Quantum Conductance in Silver Nanowires: Correlation between Atomic Structure and Transport Properties. *Phys Rev B - Condens Matter Mater Phys* **2002**, *65* (15), 1–4. https://doi.org/10.1103/PhysRevB.65.153402.

(3) Evers, F.; Korytár, R.; Tewari, S.; Van Ruitenbeek, J. M. Advances and Challenges in Single-Molecule Electron Transport. *Rev Mod Phys* **2020**, *92* (3), 35001. https://doi.org/10.1103/RevModPhys.92.035001.

(4) Ahlrichs, R.; Bär, M.; Häser, M.; Horn, H.; Kölmel, C. Electronic Structure Calculations on Workstation Computers: The Program System Turbomole. *Chem Phys Lett* **1989**, *162* (3), 165–169. https://doi.org/10.1016/0009-2614(89)85118-8.

(5) Balasubramani, S. G.; Chen, G. P.; Coriani, S.; Diedenhofen, M.; Frank, M. S.; Franzke, Y. J.; Furche, F.; Grotjahn, R.; Harding, M. E.; Hättig, C.; Hellweg, A.; Helmich-Paris, B.; Holzer, C.; Huniar, U.; Kaupp, M.; Marefat Khah, A.; Karbalaei Khani, S.; Müller, T.; Mack, F.; Nguyen, B. D.; Parker, S. M.; Perlt, E.; Rappoport, D.; Reiter, K.; Roy, S.; Rückert, M.; Schmitz, G.; Sierka, M.; Tapavicza, E.; Tew, D. P.; Van Wüllen, C.; Voora, V. K.; Weigend, F.; Wodyński, A.; Yu, J. M. TURBOMOLE: Modular Program Suite for Ab Initio Quantum-Chemical and Condensed-Matter Simulations. *J Chem Phys* **2020**, *152* (18). https://doi.org/10.1063/5.0004635.

(6) Weigend, F.; Häser, M.; Patzelt, H.; Ahlrichs, R. RI-MP2: Optimized Auxiliary Basis Sets and Demonstration of Efficiency. *Chem Phys Lett* **1998**, *294* (1–3), 143–152. https://doi.org/10.1016/S0009-2614(98)00862-8.

(7) Weigend, F.; Ahlrichs, R. Balanced Basis Sets of Split Valence, Triple Zeta Valence and Quadruple Zeta Valence Quality for H to Rn: Design and Assessment of Accuracy. *Phys Chem Chem Phys* **2005**, *7* (18), 3297–3305. https://doi.org/10.1039/b508541a.

(8) Perdew, J. P.; Burke, K.; Ernzerhof, M. Generalized Gradient Approximation Made Simple. *Phys Rev Lett* **1996**, *77* (18), 3865–3868. https://doi.org/10.1103/PhysRevLett.77.3865.

(9) Fletcher., R. *Practical Methods of Optimization*, 2ndedition ed.; Wiley: New York, 2013.

(10) Pierre Villars (Chief Editor). ,PAULING FILE in: Inorganic Solid Phases, SpringerMaterials (Online Database), Springer, Heidelberg (Ed.) SpringerMaterials Ag, Cubic (Ag) Crystal Structure. https://doi.org/https://materials.springer.com/isp/crystallographic/docs/sd_1822504.

(11) Arnold, A.; Weigend, F.; Evers, F. Quantum Chemistry Calculations for Molecules Coupled to Reservoirs: Formalism, Implementation, and Application to Benzenedithiol. *J Chem Phys* **2007**, *126* (17). https://doi.org/10.1063/1.2716664.

(12) Wilhelm, J.; Walz, M.; Stendel, M.; Bagrets, A.; Evers, F. Ab Initio Simulations of Scanning-Tunneling-Microscope Images with Embedding Techniques and Application to C58-Dimers on Au(111). *Phys Chem Chem Phys* **2013**, *15* (18), 6684–6690. https://doi.org/10.1039/c3cp44286a.

(13) Bagrets, A. Spin-Polarized Electron Transport across Metal-Organic Molecules: A Density Functional Theory Approach. *J Chem Theory Comput* **2013**, *9* (6), 2801–2815. https://doi.org/10.1021/ct4000263.
